# Evolving Trends in the Management of Duodenal Leaks After Pancreas Transplantation: A Single-Centre Experience

**DOI:** 10.3389/ti.2024.13302

**Published:** 2024-09-23

**Authors:** Samrat Ray, Christian Hobeika, Andrea Norgate, Zaneta Sawicka, Jeffrey Schiff, Gonzalo Sapisochin, Ian D. McGilvray, Markus Selzner, Trevor W. Reichman, Chaya Shwaartz

**Affiliations:** ^1^ Ajmera Transplant Centre, Toronto General Hospital, University Health Network, Toronto, ON, Canada; ^2^ Department of Surgery, University of Toronto, Toronto, ON, Canada; ^3^ Department of HPB Surgery and Liver Transplantation, Beaujon Hospital, APHP, Paris-Cité University, Clichy, France; ^4^ Division of Nephrology, Department of Medicine, University of Toronto, Toronto, ON, Canada

**Keywords:** duodenal leaks, pancreas transplantation, complications after transplantation, graft preservation, graft salvage

## Abstract

Duodenal leaks (DL) contribute to most graft losses following pancreas transplantation. However, there is a paucity of literature comparing graft preservation approach versus upfront graft pancreatectomy in these patients. We reviewed all pancreas transplants performed in our institution between 2000 and 2020 and identified the recipients developing DL to compare based on their management: percutaneous drainage vs. operative graft preservation vs. upfront pancreatectomy. Of the 595 patients undergoing pancreas transplantation, 74 (12.4%) developed a duodenal leak with a median follow up of 108 months. Forty-five (61%) were managed by graft preservation strategies, with the rest being treated with upfront graft pancreatectomy. DL managed by graft preservation strategies had similar graft survival rates at 1 and 5-year compared to the matched cohort of population without DL (95% and 59% vs. 91% and 62%; p = 0.78). Multivariate analysis identified male recipient (OR: OR: 6.18; CI95%: 1.26–41.09; p = 0.04) to have higher odds of undergoing an upfront graft pancreatectomy. In appropriately selected recipients with DL, graft preservation strategies utilizing either interventional radiology guided percutaneous drainage or laparotomy with/without repair of leak can achieve comparable long-term graft survival rates compared to recipients without DL.

## Graphical Abstract

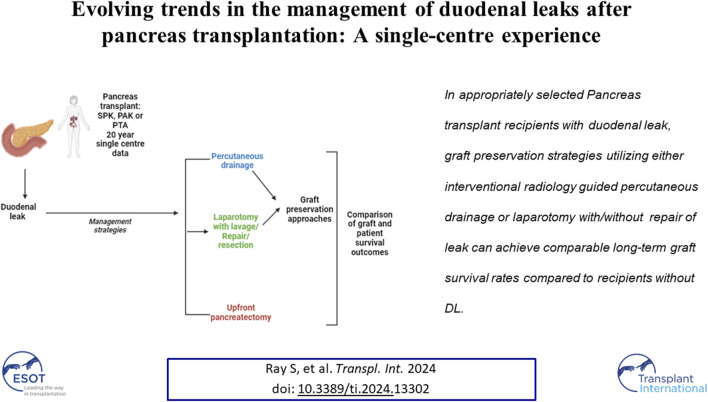

## Introduction

Recent studies have demonstrated pancreas transplantation to be the only effective method to restore euglycemia by normalizing HbA1c levels over a stable course of time following surgery [1–3]. In addition, it also improves the survival of patients with end-stage diabetic nephropathy, compared to kidney transplantation alone [4]. With advances in surgical techniques, immunosuppressive regimen, and donor-recipient selection criteria, there has been a significant improvement in the 5- and 10-year graft survival rates over the last 2 decades [5, 6]. Despite this, duodenal leaks (DL) continue to be an important complication in the setting of pancreas transplantation, with an incidence reported as high as 5%–10% in the literature, resulting in graft loss in more than 50% of those cases [7]. In most cases, the conventional treatment for graft duodenal leaks following pancreas transplantation has been laparotomy with upfront graft pancreatectomy, inevitably leading to graft loss. However, there is a paucity of literature on the efficacy of graft preservation techniques in managing duodenal leaks following pancreas transplantation.

In addition to the radical approach of upfront graft pancreatectomy for patients with graft duodenal leaks, more conservative graft-saving approaches have been increasingly employed in our institutional practice over the last 5–10 years. These approaches include interventional radiology (IR) guided percutaneous drain placement, as well as laparotomy with lavage or repair of the duodenal leak. We aimed to analyze the short- and long-term outcomes of these recipients in our 20-year cohort of pancreas transplantation and compare their results based on the management approach: upfront graft pancreatectomy versus graft-preserving strategies. The secondary objective of this study was to identify the peri-operative characteristics of these patients with duodenal leaks to determine the most appropriate initial management approach from the available options.

## Patients and Methods

### Study Population

All consecutive patients who underwent pancreas transplantation: Simultaneous pancreas-kidney (SPK), Pancreas after kidney (PAK) and Pancreas transplant alone (PTA), between January 2000 and December 2020 at the Toronto General Hospital, University Health Network were included in this study. Patients who underwent pancreas transplantation as part of a multi-visceral transplantation were excluded. All patients included in the study had a minimum of 12 months follow-up following transplantation. The study was reviewed by the ethical board (REB) of the Toronto General Hospital and approved for the study period (CAPCR ID: 21-6151.1) and adhered to the methodologic guidance from the STROBE statement [8]. The presenting complaints, physical findings, and relevant investigations (blood work, cultures, and imaging) were analyzed retrospectively from a prospectively collected transplant database. Donor and recipient demographic and peri-operative data were collected using the institutional electronic patient records database.

### Duodenal Leak

Duodenal leak (DL) was suspected in recipients presenting with fever, hyperamylasemia, elevated leucocyte count, or abdominal pain along with fluid and free air adjacent to the graft duodenum on imaging (by CT scan); the diagnosis was confirmed upon surgical exploration or imaging-guided percutaneous drainage (elevated drain fluid amylase levels: more than 3 times the serum amylase at the corresponding time point). Duodenal leaks were categorized into three groups based on their management modality: IR guided percutaneous drainage, laparotomy without pancreatectomy (lavage or repair of leak), and upfront graft pancreatectomy according to the modality of their DL management. The first two constituted the graft preserving or graft salvage approaches for management of duodenal leaks. DL patients in the first group were treated using intravenous antibiotics, fluids, nutritional support, and other supportive measures alongside percutaneous drainage of collections under interventional radiology (IR) guidance. DL patients who underwent laparotomy without graft pancreatectomy had a laparotomy with lavage and drainage with or without definitive leak repair. DL patients in the third group, who underwent upfront graft pancreatectomy had a laparotomy with resection of the graft at the time of index presentation.

### Surgical Procedures

All organ recoveries and transplantations were performed according to the standard institutional protocol with systemic venous drainage and enteric drainage of exocrine pancreas secretion described previously by our group [9]. Briefly, at the back-table preparation, the duodenal segment was shortened, ensuring adequate vascularity of the graft adjacent to the staple line, which was routinely inverted with a Lembert suture. Systemic venous drainage to the vena cava and exocrine drainage to a Roux-en-Y limb of the jejunum was routinely performed. The duodenal-jejunal anastomosis was performed in a 2-layer hand-sewn fashion and was approximately 2-3 cm long. The final orientation of the graft was behind the right colon (retro-colic), with the head up and tail towards the pelvis. A drain was left adjacent to the graft in all patients.

Intraoperative systemic anticoagulation was employed in recipients undergoing PAK or PTA only. The kidney transplant was performed before the pancreas transplant in all cases of SPK. Prophylactic antibiotics included IV cefazolin and Metronidazole before skin incision. Pre-transplant peritoneal dialysis (PD) cell count and culture sensitivity were assessed in all Kidney-pancreas (KP) patients on peritoneal dialysis as a part of the pre-transplant sepsis screen. Oral Glecaprevir/Pibrentasvir (Maviret) was administered 2–4 h before the transplant in recipients with Hepatitis C Virus (HCV) Nucleic acid testing-positive donors. Postoperative anticoagulation and antiaggregating therapy consisted of daily prophylactic with 5000 U of unfractionated heparin, and acetylsalicylic acid, 81 mg. Protocol graft ultrasound was performed on day 1 following the transplant.

### Immunosuppression

All recipients had a negative antihuman globulin complement-dependent cytotoxic T cell (before 2013) or flow cytometry crossmatch (after 2013) at the time of transplantation. Donor-specific antibodies (DSA) did not preclude transplantation, provided the crossmatch was negative. Thymoglobulin induction (3–5 mg/kg recipient body weight for SPK/PAK and up to 7 mg/kg for PTA) was administered daily over 5–7 days. Patients receiving basiliximab (Simulect) were administered an intravenous dose of 20 mg within 2 h before transplant surgery and a second dose within 12 h and on the fourth day after transplant. All patients received methylprednisolone 500 mg intraoperatively, followed by a rapid taper from 200 to 20 mg/d on day 5. The oral prednisone dosage was started at 20 mg/d, reduced to 5 mg/d at 6 months, and maintained between 2.5 and 5 mg/d thereafter. Tacrolimus (target level of 10–15 μg/L at day 7 and 5–10 μg/L at 6 months) and mycophenolate mofetil (500 mg twice a day; higher doses up to 1,000 mg BID for PTA, if tolerated) were initiated on postoperative days 2–5. Recipients with DSA also received intravenous immunoglobulin (IVIg) (1 g/kg) perioperatively.

### CMV and Other Prophylaxis

CMV-negative recipients of CMV-positive organs (Mismatch) received valganciclovir for 6 months with 6 months of monitoring post cessation of therapy, and CMV-positive recipients/CMV infection (Donor positive or negative) received 3 months of therapy. In high-risk patients (i.e., CMV-positive organ to CMV-naive recipients), CMV viremia was monitored by quantitative polymerase chain reaction for 3 months after the cessation of valganciclovir; a 6-week course of valganciclovir was started in those patients who became viremic. Ganciclovir’s initial dose was 5 mg/kg IV daily, followed by an oral dose of 1 g three times a day or 900 mg of oral valganiclovir per day whenever the patient could tolerate oral medications. Acyclovir 400 mg BID prophylaxis for 3 months was given in CMV-negative recipients with negative donors. Pneumocystis carinii pneumonia (PCP) prophylaxis was started in all recipients (Cotrimoxazole Single strength alternate day).

### Follow-Up and Survival Endpoints

Duration of follow-up and outcomes of interest such as postoperative complications (arterial/venous/systemic), 90-day post-transplant mortality, infections (bacterial, viral, and fungal), rejection episodes (pancreas/kidney/both; pancreas rejections classified by the Maryland system) [10] pancreas graft failure (defined as a return to insulin dependency), cause of graft failure and loss, and death on follow-up were collected. Patients in the percutaneous drainage and laparotomy groups requiring graft pancreatectomy at any point during the study period were considered to have experienced graft loss. Similarly, re-laparotomy during the study period was recorded as a separate event in the postoperative outcomes for the three groups. Graft survival (GS) was defined as the time from transplantation to graft failure (pancreas alone or combined pancreas-kidney). Overall survival (OS) was defined as the time from transplantation to the time of death (from any cause). Duodenal leak-free survival (DLFS) was defined as the months survived without a duodenal leak after the index pancreas transplantation.

### Statistical Analysis

Continuous data were expressed as median (25–75 inter-quartiles), were not categorized, and were compared using the Mann-Whitney *U* test or Kruskal-Wallis test, as appropriate. Categorical data are expressed as percentages and were compared using Pearson’s chi-square test or Fisher’s exact test, as appropriate. Statistical significance testing was 2-sided. Unless indicated otherwise, a p-value <0.05 was considered statistically significant for all tests. There were no missing values regarding the endpoints of this study, including the events of duodenal leakage, recurrence, or death, and the times to duodenal leakage, recurrence, or death, and no variable had more than 10% of data missing. Continuous variables were transformed in the regressions using natural splines with three degrees of freedom to avoid non-linear relationship misspecification due to the non-normal distribution of biological data. Survival probabilities were computed using the Kaplan-Meier estimate and compared using the log-rank test.

Duodenal leakage was encoded as a time-dependent variable (i.e., right censored outcome). Right-censored outcomes (i.e., DLFS, OS and DFS) regressions were analyzed using Cox proportional-hazards regression models [estimated effect sizes were expressed as Hazard Ratio (HR) with 95% confidence interval (CI95%)]. To include time-dependent covariates in the Cox model, the dataset was transformed into a long format (i.e., multiple observations at each time point for one individual), and each observation was correlated to each individual using a cluster term in Cox models. Binary outcomes regressions were logistic regressions (estimated effect sizes were expressed as Odd’s Ratio (OR) with CI95%). Dimensional reduction of models was performed using a semi-automated stepwise backward-forward selection of variables based on Akaike information criteria [11]. To enhance the clinical consistency of our reduced model, a set of preoperative variables clinically relevant for capturing patient profiles in pancreatic transplantation was considered as follows: recipient age, BMI and sex as well as the type of donor, cold ischemia time, and type of transplant. The reduced models included these variables regardless of whether they were selected. Additionally, the variable of interest for this study (i.e., DL) was also forced in models when appropriate (i.e., in the survival analyses).

A model of exposure was estimated using a propensity score matching (PSM), which was performed using a 5:1 (5 controls matched to 1 case) nearest neighbor matching without replacement. A propensity score was estimated with logistic regression, including clinically relevant variables or variables associated with the exposure/outcome in the exploratory analysis as follows: age, BMI, CMV status of the donor and the recipient, modality pancreatic transplantation (PTA, SPK, PAK), the type of donor (DCD vs. DBD), cold ischemia time, postoperative pulmonary or septic related complication, requirement for dialysis after transplant, graft portal vein thrombosis and rejection. Covariate balance before and after matching were estimated using standardized mean differences (see Supplementary Figure S1). The marginal effect of DL on survivals in the matched population was estimated using a weighted (incorporating the matching weights) Cox model without covariates (i.e., non-collapsible HR) with clustered variance on matching pair membership (cluster-robust standard errors).

All statistical analyses were performed using R statistical software version R version 4.2.0.

## Results

### Study Population

A total of 595 patients underwent pancreas transplantation during study period, the majority being SPK (72.8%; n = 433), followed by PAK (23.5%; n = 140) and PTA (3.7%; n = 22). Among them, 63.2% (n = 376) were males, and the median age (IQR) and BMI (IQR) were 43.5 years (37.2–50.5) and 24.7 kg/m^2^ (21.8–27.9), respectively. The rate of 90-day mortality was 5% (n = 30). The descriptive analysis of the whole population and comparison between patients with and without DL is shown in Table 1.

**TABLE 1:** Demographic, pre-operative and peri-operative characteristics of Duodenal leak (DL) group compared with the control group of patients in overall cohort of pancreas transplantation recipients.

Variables	Overall (n = 595)	DL (n = 74)	Control (n = 521)	P-value
Donor Age (years) (IQR)	25 (19–34)	23.5 (18–36)	25 (19–34)	0.74
DCD donors (%)	31 (5.2)	2 (2.7)	29 (5.5)	0.03
Donor BMI (kg/m^2^) (IQR)	23.2 (20.8–26.1)	24.3 (19.9–27.5)	23.1 (21–25.9)	0.48
Donor WIT (DCD; mins) (IQR)	26 (22.5–28)	10 (10–10)	26 (24–28)	0.02
Donor CIT (mins) (IQR)	542 (442.2–644.7)	527.5 (440.5–615.5)	545 (443.5–647)	0.19
Recipient Age (years) (IQR)	43.5 (37.3–50.5)	42 (36.7–47)	43.9 (37.4–50.7)	0.16
Recipient gender (% male)	376 (63.2)	47 (63.5)	329 (63.1)	>0.99
Recipient BMI (kg/m^2^) (IQR)	24.7 (21.8–27.9)	25.8 (22.6–29.1)	24.6 (21.6–27.7)	0.02
Recipient CMV status				0.82
CMV Mismatch (D+/R-) (%)	86 (14.5)	12 (16.2)	74 (14.2)	
CMV infection (R+) (%)	65 (10.9)	9 (12.2)	56 (10.7)	
Recipient EBV status				0.53
EBV Mismatch (D+/R-) (%)	27 (4.5)	2 (2.7)	25 (4.8)	
EBV infection (R+) (%)	4 (0.7)	0 (0)	4 (0.8)	
Transplant category				0.42
SPK (%)	433 (72.8)	53 (71.6)	380 (72.9)	
PAK (%)	140 (23.5)	20 (27)	120 (23)	
PTA (%)	22 (3.7)	1 (1.4)	21 (4)	
Pre-transplant IS(%)	40 (6.7)	4 (5.4)	36 (6.9)	0.81
Pre-transplant Dialysis (%)	215 (36.1)	25 (33.8)	190 (36.5)	0.75
Pre-transplant cardiac intervention (%)	215 (36.1)	27 (36.5)	188 (36.1)	>0.99
Pre-transplant infections (%)	53 (8.9)	8 (10.8)	45 (8.6)	0.69
Post-transplant dialysis (%)	25 (4.2)	4 (5.4)	21 (4)	0.81
Post-transplant DVT (%)	5 (0.8)	1 (1.4)	4 (0.8)	>0.99
Post-transplant pneumonia (%)	14 (2.4)	3 (4.1)	11 (2.1)	0.53
Post-transplant CLABSI (%)	1 (0.2)	0 (0)	1 (0.2)	>0.99
Post-transplant stay (Days) (IQR)	9.6 (8–13.2)	10.7 (8.6–18.7)	9.6 (7.8–12.7)	0.01
Graft related complications				
Arterial thrombosis (%)	3 (0.5)	1 (1.4)	2 (0.4)	0.82
Portal vein thrombosis (%)	15 (2.5)	2 (2.7)	13 (2.5)	>0.99
Hemorrhage (%)	27 (4.5)	5 (6.7)	22 (4.2)	0.61
Graft rejection (pancreas) (%)	74 (12.4)	12 (16.2)	62 (11.9)	0.41
Graft rejection (Kidney) (%)	36 (6.1)	12 (16.2)	24 (4.6)	0.06
Graft loss (pancreas) (%)	150 (25.2)	35 (47.3)	115 (22.1)	<0.001
Graft loss (Kidney) (%)	53 (8.9)	9 (12.1)	44 (8.4)	0.78
Re-transplantation (pancreas) (%)	42 (7.1)	15 (20.3)	27 (5.2)	<0.001
Overall mortality (%)	112 (18.8)	14 (18.9)	98 (18.8)	>0.99

** All continuous variables expressed as medians, unless specified otherwise.

Legends: DL, Duodenal leak; IQR, Interquartile range; DCD, Donation after cardiac death; BMI, Body mass index; WIT, Warm ischemia time; CIT, Cold ischemia time; CMV, Cytomegalovirus; EBV, Epstein Barr Virus; D/R, Donor/Recipient; SPK, Simultaneous pancreas kidney; PAK, Pancreas after kidney; PTA, Pancreas transplant alone; IS, Immunosuppression; DVT, Deep venous thrombosis; CLABSI, Central line associated bloodstream infections.

### DL Occurrence After Pancreatic Transplantation

**FIGURE 1:**
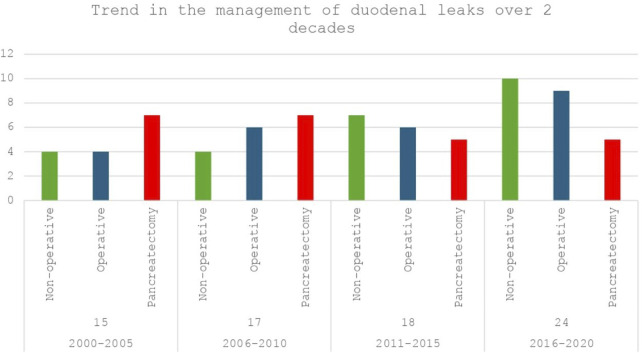
Trend of management of duodenal leaks (DL) after pancreas transplantation at the Toronto general hospital over 2 decades (2000–2020): Non-operative (IR guided drain placement) vs. Operative (Laparotomy with lavage/repair) vs. Upfront pancreatectomy.

After a median follow-up of 108 months, 74 patients (12.4%) were found to develop DL, with 42% (n = 31) developing leaks within 90 days following transplantation. DL patients underwent management with IR guided percutaneous drain insertion, laparotomy without graft pancreatectomy (lavage/repair), and upfront graft pancreatectomy in 29.7% (n = 22), 31.1% (n = 23), and 39.2% (n = 29) of the cases. A comparison of the demographic and peri-operative characteristics between the three groups is summarised in Supplementary Table S1. Figure 1 depicts a 5-yearly comparative trend of the 3 management modalities for DL during the study period (2000-20).

### Influence of DL on Overall and Graft Survivals

**FIGURE 2:**
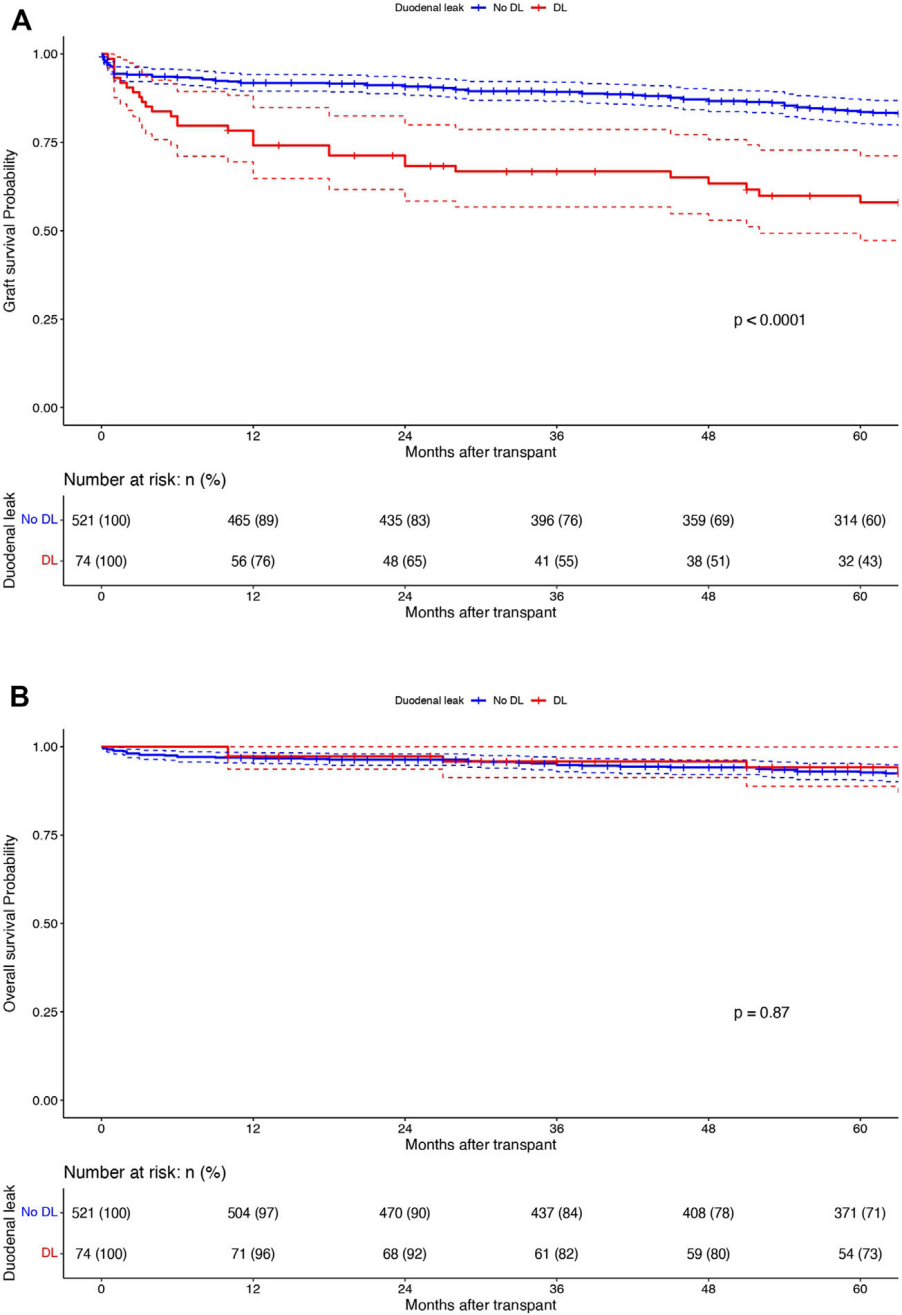
Kaplan-Meier curves displaying **(A)** graft survivals and **(B)** overall survivals in patients with (n = 74; red) and without DL (n = 521; blue). X-axis: months after transplant. Y-axis: survival probabilities. Comparison using Log-rank test.

In the whole population (n = 595), 1-, 3- and 5-year overall and graft survivals were 96.8%, 95% and 92.9% and 89.6%, 86.5%, and 80.4%, respectively. One-, 3- and 5 years DLFS were 91%, 88.8%, and 86.8%, respectively. DL patients were associated with a decreased 1-/3-/5-year graft survival (74.2%/66.8%/58% vs. 91.9%/89.3%/83.6% respectively; p < 0.0001) while they had similar 1-/3-/5-year OS (96.5%/93.8%/92.4% vs. 96.7%/94.2%/92.7% respectively; p = 0.87) when compared to the patients without DL (Figures 2A, B). On multivariable analysis by Cox proportional hazards model, DL was independently associated with decreased graft survival (HR: 3.45; CI95%: 2.10–5.68; p < 0.001) but not with OS (HR: 0.38; CI95%: 0.05–2.89; p = 0.36). Tables 2, 3; Supplementary Table S2 report reduced and full Cox models to assess graft survival and overall survival risk factors in the population (n = 595).

**TABLE 2:** Cox proportional hazards model in the whole population (n = 595) to identify factors associated with graft survival (GS) (Reduced model).

Variables	Graft survival		
	HR	95% CI	P-value
Recipient age (years)^a^			
Spline for age ≤37.3 years	0.66	0.42–1.08	0.06
Spline for age >37.3 and ≤50.5 years	0.68	0.40–1.08	0.39
Spline for age >50.5 years	0.78	0.43–1.43	0.35
Recipient BMI (kg/m [2])^a^			
Spline for BMI ≤21.8 kg/m [2]	0.95	0.50–1.79	0.85
Spline for BMI >21.8 and ≤27.9 kg/m [2]	1.06	0.58–1.93	0.83
Spline for BMI >27.9 kg/m [2]	1.08	0.55–2.10	0.80
Male recipient	1.27	0.87–1.85	0.15
CMV status			
Negative	ref	ref	ref
CMV infection (R+)	1.38	0.86–2.21	0.08
CMV mismatch (D+/R-)	1.16	0.73–1.85	0.42
Transplant category			
Simultaneous pancreas kidney	ref	ref	ref
Pancreas after kidney	1.48	1.01–2.16	0.01
Pancreas transplant alone	1.53	0.54–4.34	0.35
Pre-transplant infection	1.58	1.15–2.65	0.02
Donor type (DCD vs. DBD)	1.27	0.46–3.52	0.59
Donor CIT (mins)^a^			
Spline for CIT ≤442.2 min	1.09	0.61–1.93	0.73
Spline for CIT >442.2 and ≤644.7 min	1.13	0.67–1.92	0.57
Spline for CIT >644.7 min	1.12	0.64–1.97	0.63
Post transplant dialysis	1.87	1.04–3.37	0.006
Graft PV thrombosis	1.67	0.64–4.03	0.18
Duodenal leak	3.45	2.10–5.68	<0.001
Graft rejection (pancreas)	1.62	1.07–2.45	0.004

Abbreviations: GS, graft survival; HR, hazard ratio; CI, confidence interval; BMI, body mass index; CMV, cytomegalovirus; DCD, donation after cardiac death; DBD, donation after brain death; CIT, cold ischemia time; PV, portal vein.

^a^ Non-linear variables transformed using cubic spline functions with 3 degrees of freedom; knots are placed at the 25th and 75th percentiles of the variable in the overall population.

**TABLE 3:** Cox proportional hazards model in the whole population (n = 595) to identify factors associated with overall survival (OS) (Reduced model).

Variables	OS		
	HR	95% CI	P val
Recipient age (yrs)			
Spline for age ≤37.3 years	1.12	0.49–2.55	0.85
Spline for age>37.3 and ≤50.5 years	1.26	0.96–4.81	0.09
Spline for age >50.5 years	4.06	1.78–9.30	0.008
Recipient BMI (kg/m [2])			
Spline for BMI ≤21.8 kg/m [2]	0.49	0.26–0.93	0.04
Spline for BMI >21.8 and ≤27.9 kg/m [2]	0.51	0.28–0.93	0.03
Spline for BMI >27.9 kg/m [2]	0.82	0.43–1.56	0.56
Male recipient	0.77	0.55–1.19	0.25
CMV status			
Negative	ref	ref	ref
CMV infection (R+)	0.79	0.41–1.53	0.49
CMV mismatch (D+/R-)	0.99	0.49–2.00	0.98
Transplant category			
SPK	ref	ref	ref
PAK	0.98	0.59–1.63	0.95
PTA	0.77	0.18–3.34	0.73
Donor type (DCD vs. DBD)	0.69	0.25–1.89	0.52
Donor CIT (mins)			
Spline for CIT ≤442.2 min	0.74	0.38–1.44	0.39
Spline for CIT >442.2 and ≤644.7 min	1.14	0.63–2.05	0.67
Spline for CIT >644.7 min	1.12	0.59–2.13	0.73
Post transplant pneumonia	4.02	1.63–9.89	<0.001
Duodenal leak	0.38	0.05–2.89	0.36

Legends: OS, overall survival; HR, hazard ratio; CI, confidence interval; BMI, body mass index; CMV, cytomegalovirus; SPK, simultaneous pancreas kidney; PAK, pancreas after kidney; PTA, pancreas transplant alone; DCD, donation after cardiac death; DBD, donation after brain death; CIT, cold ischemia time.

*Non-linear variables transformed using cubic spline functions with 3 degrees of freedom; knots are placed at the 25th and 75th percentiles of the variable in the overall population.

### Management of DL (Comparison of the 3 Groups of Management of DL)

**FIGURE 3:**
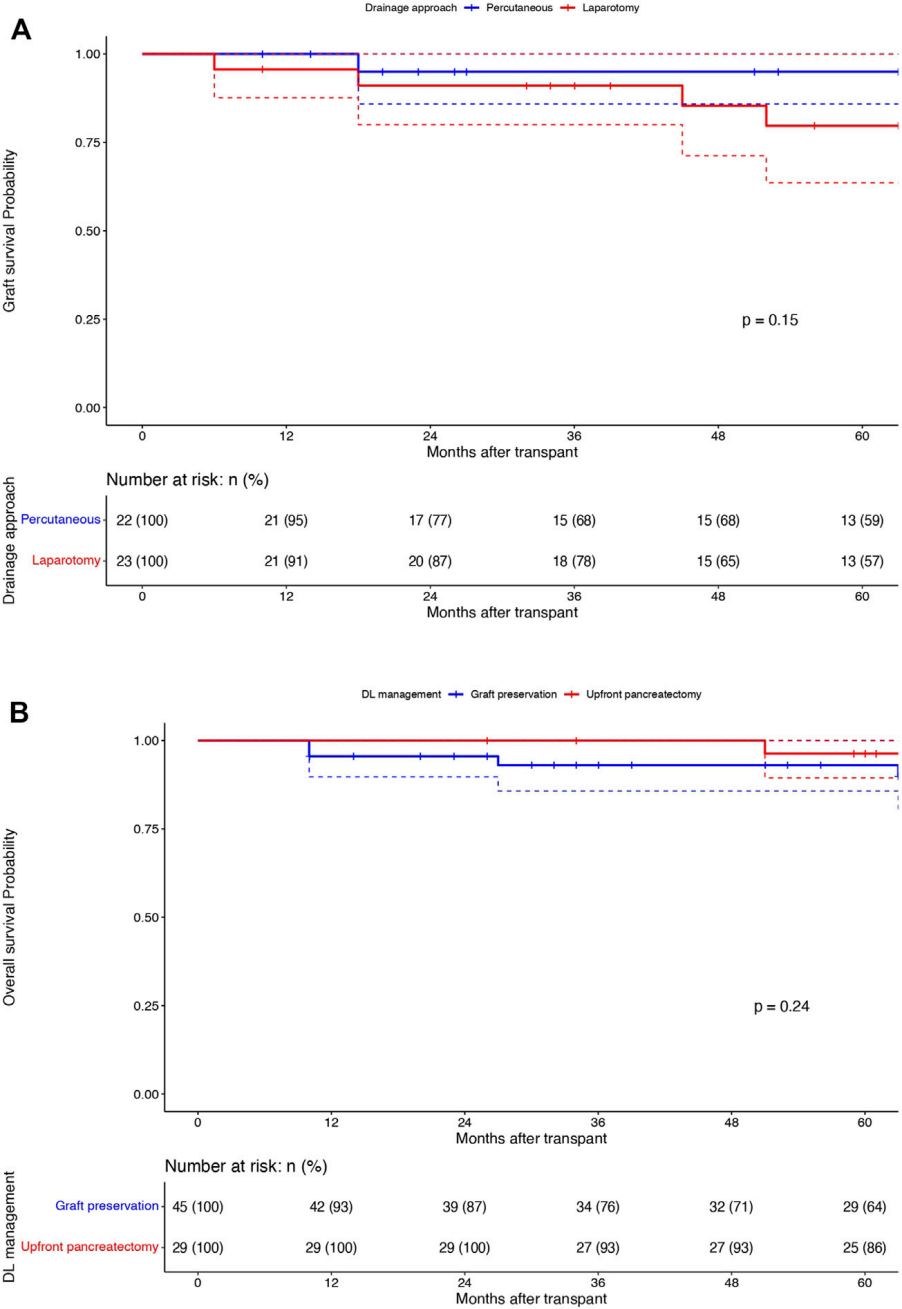
Graft survival (1/3/5 years): Percutaneous drainage (Blue) vs. Laparotomy (Red) without pancreatectomy groups: X-axis: Months after transplant and Y-axis: Graft survival probability; comparison using Log rank test **(A)**. Overall survival (1/3/5 years): Graft preservation (Blue) vs. pancreatectomy (Red) groups: X-axis: Months after transplant and Y-axis: overall survival probability; comparison using Log rank test **(B)**.

Among the DLs managed by graft salvage strategies, there was no difference in the graft survival between the percutaneous drainage arm (n = 22) and the laparotomy without pancreatectomy arm (n = 23) (95%/68%/59% vs. 91%/78%/57%; p = 0.15) (Figure 3A). The comparison of overall patient survival curves is demonstrated in Figure 3B. The overall survival rates were comparable between the graft salvage and graft pancreatectomy groups. Besides this, in the cohort of DLs managed by percutaneous drainage alone, 2 out of 22 required a laparotomy, in view of persistent undrainable collections and hemodynamic worsening. The mean interval between drain placement to removal was 17.5 days (12–53) and 8 out of 22 patients (36.3%) required repeat drain placement during the study period. In the laparotomy without pancreatectomy group, 3 out of 23 patients required re-laparotomy, with 2 culminating into graft pancreatectomy eventually. Although we observed a higher rate of renal rejection in DLs managed by graft preservation approaches compared to upfront pancreatectomy (22.7% and 17.4% vs. 10.3%), this was not significant and was managed by appropriate immunosuppressive therapy in all cases.

### Matching Comparison of DL Patients (Without Upfront Graft Pancreatectomy)

**FIGURE 4:**
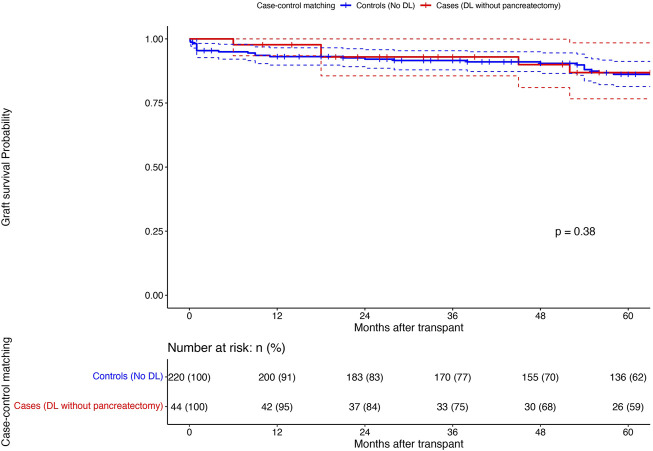
Comparison of 1/3/5 years pancreas graft survival (Kaplan-Meier curves; Log rank test) between the DL patients without upfront pancreatectomy (n = 44; Cases; Red) and a matched (1:5) population of patients without DL (n = 220; Controls; Blue): X-axis: Months after transplant and Y-axis: Graft survival probability; comparison using Log rank test.

Among the DL group, 44 patients who did not undergo upfront graft pancreatectomy (cases) were matched (1:5) to patients without DL (controls) (Propensity score matching; see Supplementary Figure S1 for details). Table 4 shows the matched population’s comparative analysis between cases and controls. The marginal effect of DL on graft survival was null (HR:0.00, 95%CI:0.00–0.00; p < 0.001), with no difference in the 1/3/5-year graft survival between the cases and the matched controls (95%/75%/59% vs. 91%/77%/62%; p = 0.87) (Figure 4).

**TABLE 4:** Comparison of the demographic, pre-operative and peri-operative characteristics between the matched population of duodenal leak cohort without upfront pancreatectomy (graft preservation group) (n = 44) with the control population (n = 220); Propensity score matching (DL: Control = 1:5).

Variables	Matched control (n = 220)	DL without pancreatectomy (n = 44)	P val
Donor age (yrs) (IQR)	25.5 (20–35)	26 (19–36)	0.95
Donor BMI (kg/m^2^) (IQR)	23.2 (20.9–26.1)	24.5 (20–27.1)	0.08
DCD donors (%)	10 (4.5)	2 (4.5)	>0.99
Donor CIT (mins) (IQR)	517 (414.5–601)	504 (405.2–600)	0.87
Recipient age (yrs) (IQR)	42.5 (37.4–48.4)	42.2 (38.4–46.8)	0.89
Recipient BMI (kg/m^2^) (IQR)	25.8 (22.5–28.9)	25.9 (23.4–29.6)	0.67
Recipient gender (% males)	147 (66.8)	25 (56.8)	0.27
CMV mismatch (%)	20 (9.1)	4 (9.1)	0.24
EBV mismatch (%)	12 (5.5)	0 (0)	0.23
Transplant category			
SPK (%)	170 (77.3)	34 (77.3)	>0.99
PAK (%)	45 (20.5)	9 (20.5)	
PTA (%)	5 (2.3)	1 (2.3)	
Pre-transplant IS (%)	14 (6.4)	0 (0)	0.17
Pre-transplant Dialysis (%)	90 (40.9)	14 (31.8)	0.34
Pre-transplant cardiac intervention (%)	86 (39.1)	14 (31.8)	0.46
Pre-transplant infections (%)	23 (10.5)	5 (11.4)	>0.99
Post-transplant dialysis (%)	11 (5)	2 (4.5)	>0.99
Post-transplant DVT (%)	0 (0)	1 (2.3)	0.24
Post-transplant pneumonia (%)	6 (2.7)	1 (2.3)	>0.99
Post-transplant CLABSI (%)	1 (0.5)	0 (0)	>0.99
Post-transplant stay (days) (IQR)	9.7 (8.5–12.7)	10.1 (8.6–20.9)	0.31
Graft related complications			
Arterial thrombosis (%)	1 (0.5)	1 (2.3)	0.75
Portal vein thrombosis (%)	9 (4.1)	2 (4.5)	>0.99
Hemorrhage (%)	10 (4.5)	3 (6.8)	0.45
Graft rejection (pancreas) (%)	31 (14.1)	7 (15.9)	0.94
Graft loss (pancreas) (%)	45 (20.5)	6 (13.6)	0.40
Re-transplantation (pancreas) (%)	9 (4.1)	3 (6.8)	0.69
Overall mortality (%)	35 (15.9)	5 (11.4)	0.59
Median DLFS (months) (IQR)	84.5 (39.5–142.2)	12 (2.0–24.0)	**<0.001**
Median OS (months) (IQR)	99.5 (51.7–162)	83.5 (38.2–162.7)	0.55

** All continuous variables expressed as medians, unless specified otherwise.

Legends: DL, duodenal leak; IQR, interquartile range; BMI, body mass index; DCD, donation after cardiac death; CIT, cold ischemia time; CMV, cytomegalovirus; EBV, epstein barr virus; D/R: Donor/Recipient; SPK, simultaneous pancreas kidney; PAK, pancreas after kidney; PTA, pancreas transplant alone; IS, immunosuppression; DLFS, duodenal leak free survival; OS, overall survival.

The bold values indicate statistical significance.

### Identifying the High-Risk Factors in DL Cohort (Predictors of Upfront Graft Pancreatectomy)

The multivariable analysis (reduced model) identified presence of male recipient (OR:6.18; CI95%: 1.26–41.09; p = 0.04) to be associated with higher odds of requiring an upfront graft pancreatectomy on reduced model. Presence of CMV infection in the recipient (OR: 17.8; 95% CI:4.10–5,720; p = 0.02) and pre-transplant cardiovascular intervention (OR:10.8; 95%CI: 1.57–108; p = 0.03) were other variables associated with relatively higher odds of upfront pancreatectomy, although not found significant on reduced model (Table 5).

**TABLE 5:** Logistic regression model to identify high-risk predictors in duodenal leak cohort (associated with upfront pancreatectomy) (Full and reduced regression model).

Variables	Full model			Reduced model		
	OR	95% CI	P val	OR	95% CI	P val
Donor age (yrs)						
Spline for age ≤19 years	0.61	0.00–181	0.87	0.62	0.06–5.32	0.66
Spline for age >19 and ≤34 years	0.02	0.00–1.47	0.13	0.07	0.00–0.81	0.04
Spline for age >34 years	4.20	0.01–3,607	0.63	1.64	0.15–19.41	0.68
Donor BMI (kg/m [2])						
Spline for BMI ≤20.8 kg/m [2]	0.02	0.00–1.58	0.15			
Spline for BMI >20.8 and ≤26.1 kg/m [2]	0.32	0.00–18.2	0.57			
Spline for BMI >26.1 kg/m [2]	0.32	0.00–113	0.72			
DBD vs. DCD donor	76.2	0.00–189	0.99			
Donor CIT (mins)						
Spline for CIT ≤442.2 min	31.1	0.12–840	0.33	4.80	0.36–109.68	0.26
Spline for CIT >442.2 and ≤644.7 min	6.29	0.04–120	0.57	4.17	0.44–73.75	0.25
Spline for CIT >644.7 min	0.79	0.00–87	0.95	7.50	0.69–156.43	0.13
Recipient age (yrs)						
Spline for age ≤37.3 years	1.89	0.00–2,976	0.84	0.94	0.12–7.70	0.95
Spline for age>37.3 and ≤50.5 years	0.01	0.00–2.42	0.13	0.24	0.03–1.78	0.16
Spline for age >50.5 years	1.50	0.01–859	0.88	1.60	0.14–23.46	0.71
Recipient BMI (kg/m [2])						
Spline for BMI ≤21.8 kg/m [2]	1.54	0.01–598	0.87	0.55	0.06–6.64	0.62
Spline for BMI >21.8 and ≤27.9 kg/m [2]	0.32	0.00–55.6	0.62	0.30	0.04–2.56	0.25
Spline for BMI >27.9 kg/m [2]	0.01	0.00–6.04	0.21	0.25	0.02–2.69	0.26
Male recipient	23.8	0.57–5,777	0.14	6.18	1.26–41.09	**0.04**
CMV status						
Negative	ref	ref	ref	ref	ref	ref
CMV mismatch (D+/R-)	24.8	0.66–5,347	0.12	4.28	0.83–25.69	0.09
CMV infection (R+)	17.8	4.10–5,720	**0.02**	2.01	0.32–12.52	0.44
EBV mismatch (D+/R-)	4.24	0.00-NA	>0.99			
Transplant category						
SPK	ref	ref	ref	ref	ref	ref
PAK	0.18	0.00–8.93	0.41	3.91	0.80–23.23	0.10
PTA	0.00	NA	>0.99	4.73e-06	--,8.04e+117	0.99
Pre-transplant IS	190	0.00–9,700	0.91	8.9	0.00–97	0.89
Pre-transplant dialysis	0.34	0.00–7.95	0.57			
Pre-transplant cardiac intervention	10.5	1.57–108	**0.03**			
Pre-transplant infection	0.15	0.00–21.9	0.51			

Legends: OR: Odd’s ratio, CI: confidence interval, DBD: donation after brain death, DCD: donation after cardiac death, BMI: body mass index, CIT: cold ischemia time, CMV: cytomegalovirus, EBV: epstein barr virus, D/R: Donor/Recipient, SPK: simultaneous pancreas kidney, PAK: pancreas after kidney, PTA: pancreas transplant alone, IS: immunosuppression.

Non-linear variables transformed using cubic spline functions with 3 degrees of freedom; knots are placed at the 25th and 75th percentiles of the variable in the overall population.

The bold values indicate statistical significance.

## Discussion

The present study demonstrated a 1-year graft loss of 25% in patients with DL, with 42% being diagnosed within the first 90 days after pancreas transplantation. Even though there was a demonstrable difference in the graft survival rate between the DL cohort and the patients without DL, we observed no difference in the overall survival rate at the corresponding time points. Further comparison between the patients with DL managed with graft preservation strategies (percutaneous drainage and laparotomy without pancreatectomy) and a matched group of patients without DL demonstrated a marginal effect of DL *per se*, on short and long-term graft survival, with comparable graft survival rates at 1-/3-/5-year. Pre-transplant sepsis, graft rejection episodes, post-transplant dialysis, graft PV thrombosis were identified as the independent predictors of poor graft survival after pancreas transplantation, besides duodenal leak. In patients with duodenal leak after pancreas transplantation, development of an early leak (within 90 days), presence of CMV mismatch (D+/R−), male recipient and pre-transplant cardiovascular intervention were found to predict a worse outcome, necessitating an upfront pancreatectomy approach in most of these patients.

Management of DLs after pancreas transplantation have traditionally leaned towards an aggressive approach involving early detection and surgical intervention, with upfront graft pancreatectomy in most cases [12, 13]. The choice of graft preserving approach versus upfront graft pancreatectomy remains a matter of debate. Graft-preserving approaches, while aiming to maintain insulin independence, are associated with high rates of readmissions, relaparotomy, kidney graft rejection and failure, and sepsis, according to the limited available literature, which mostly consists of case reports and small series [14, 15]. Al-Adra et al from our centre investigated the outcome of DLs after pancreas transplantation, comparing the graft salvage approaches to upfront graft pancreatectomy in a series of 33 recipients with DL [16]. The authors reported favourable outcomes for laparotomy with definitive repair in carefully selected patients with limited peritoneal contamination and localised source of leak (13 of 14 patients with a median graft survival of 2.9 years). However, more conservative measures such as percutaneous drainage and operative drainage by lavage failed to control the leak, necessitating graft pancreatectomy in 7 out of the 8 cases. Findings of this study were further substantiated by Fleetwood et al. in 2022 [17]. The authors compared the outcomes of graft salvage approach (repair and resection) with immediate graft pancreatectomy in a series of 33 patients with DL out of 1,153 undergoing pancreas transplantation. They found DL to be an independent predictor of 6-month graft loss (HR: 13.9; CI95%: 8.5–22.9; p < 0.001). However, they reported no difference in 5-year graft survival (82.5% vs. 81.5%) and overall survival (90.5% vs. 93.5%) between the graft salvage and non-leak groups beyond 6 months. This group did not attempt percutaneous drainage in any of their patients. Similar to the findings by the Wisconsin group [17], we observed no difference in the 5-year graft survival rates between DL patients who underwent graft salvage (conservative or operative) and recipients without DL. This was further substantiated by comparing the graft survival and other peri-operative variables between the DL with graft salvage cohort and a matched population of recipients without DL. We observed no difference in the 1- and 5-year graft survival rates (95% and 59% vs. 91% and 62%, respectively). Additionally, we noted a favorable outcome in terms of graft survival in the group treated by percutaneous drainage alone, with a median graft survival of 65 months.

A concern with the percutaneous drainage-alone management was a higher rate of re-admissions (10 out of 22 patients; 45.4%). These readmissions were primarily due to fever, elevated total leukocyte counts, ileus, or undrained collections. These complications were managed by upsizing the percutaneous drains or placing multiple drains in new collections under IR guidance. Previous studies have also discussed a theoretically higher risk of 90-day mortality with graft salvage approaches, potentially due to the development of generalized peritonitis and abdominal sepsis from persistent undrained collections, leading to severe systemic inflammation and septic shock. In our study, we found no significant difference in the 90-day mortality rates between the three groups, with a slightly higher incidence in the upfront pancreatectomy group compared to the graft preservation groups (27.6% vs. 13.6% and 13%; p = 0.76) [18, 19].

Benefits of graft salvage approaches include maintaining insulin independence and avoiding the need for re-transplantation, which is significant considering the high waitlist times and organ shortage. These benefits must be weighed against the potential risks mentioned above [20]. Therefore, triaging patients with DL into graft salvage vs. upfront graft pancreatectomy groups is important for an appropriate decision making for a multiorgan transplantation team. Clinical determinants such as hemodynamic worsening, persistently elevated blood counts, persistent undrained collections on serial imaging, and definitive evidence of unresolving sepsis or multiple undrained collections indicate the need for upfront pancreatectomy. Additionally, identification of other demographic and peri-operative factors in the donor and recipient may be helpful in decision making in these patients. We found that recipient factors such as male gender and presence of CMV infection in the recipient and pre-transplant cardiovascular intervention associated with higher odds of undergoing upfront pancreatectomy in the DL cohort, the latter two not being statistically significant. Male recipient has been shown to be associated with a higher incidence of portal vein thrombosis (higher risk of DL) in a few studies although the more recent literature has not shown any significant impact of recipient gender on the incidence of the same [21, 22]. CMV infection and pre-transplant cardiovascular interventions have been shown to be associated with delayed and very early leaks respectively, by a previous study from our centre, both of which might be presumably challenging to manage by more conservative measures due to the presence of other risk factors of healing like higher dose of immunosuppression in the early post-operative phase (in PAK and SPK) or risk of renal rejection (especially in delayed leaks) [23]. This could possibly explain the higher odds of upfront pancreatectomy (albeit not significant) in these groups of recipients in our patient population.

There were certain limitations of this study as well, foremost being the number of patients with DL. Contrary to the available literature, we observed a slightly higher incidence of DL in our cohort [23, 24]. The criteria for diagnosing DLs at our centre was mainly based on imaging using CT scan and/or drain amylase levels (in patients with percutaneous drain placement under CT guidance), the latter having a low specificity for DL. Additionally, the threshold for placing percutaneous drains in these patients has been low with a trend towards a more of a “drain first” approach over the last decade. This shift is mainly attributed to improvements in the precision of IR guidance and clinicians’ preferences for managing DLs in this manner. A preference for earlier intervention in these patients has also been observed, anticipating better outcomes in terms of graft preservation. These factors may explain the higher reporting of DLs during the 20-year study period. Another important limitation was the retrospective nature of the study. Although our results with graft salvage approaches are promising, larger multicentre analysis is needed in future to validate these findings more conclusively to account for variations in operative techniques and pre- and post-transplant management protocol of pancreas transplant recipients from centre to centre.

In conclusion, despite the seemingly devastating effects of duodenal leaks on graft survival, an early diagnosis and timely intervention can decrease the short- and long-term morbidity and mortality in these patients. Graft salvage strategies have shown promising long-term results in selected patients. Triaging patients into graft salvage versus upfront graft pancreatectomy approaches, based on a combination of peri-operative donor and recipient characteristics, clinical presentation of DL, radiological severity of peritoneal contamination, and the expertise of the IR team to target undrained collections, remains the cornerstone of management for achieving favorable outcomes in pancreas transplant recipients with duodenal leaks.

## Data Availability

The raw data supporting the conclusions of this article will be made available by the authors, without undue reservation.

## Abbreviations

DL, Duodenal leak; IR, Interventional radiology; HbA1C, Hemoglobin A1C; SPK, Simultaneous pancreas kidney; PAK, Pancreas after kidney; PTA, Pancreas transplant alone; STROBE, Strengthening the reporting of observational studies in epidemiology; IR, Interventional radiology; GIA, Gastrointestinal anastomosis; PD, Peritoneal dialysis; KP, Kidney-pancreas; HCV, Hepatitis C Virus; DSA, Donor specific antibodies; IVIg, Intravenous immunoglobulin; CMV, Cytomegalovirus; PCP, Pneumocystis carinii pneumonia; GS, Graft survival; OS, Overall survival; DLFS, Duodenal leak free survival; HR, Hazard ratio; CI95%, 95% confidence interval; OR, Odd’s ratio; PSM, Propensity score matching; BMI, Body mass index; DCD, Donation after cardiac death; DBD, Donation after brain death; IQR, Interquartile range.
