# Supplementary material for: Evolving Trends in the Management of Duodenal Leaks After Pancreas Transplantation: A Single-Centre Experience
Source: Transpl Int. 2024 Sep 23;37:13302. doi: 10.3389/ti.2024.13302 (PMC11456492; doi:10.3389/ti.2024.13302)
Supplement: Supplementary file 1 [file Table1.docx]

**Supplementary table 1:** Demographic, peri-operative and post-operative variables in the duodenal leak (DL) cohort (n=74) compared between the 3 subgroups based on the management: Percutaneous drainage (n=22), Laparotomy without pancreatectomy (n=23) and Upfront pancreatectomy (n=29).

| **Variables** | **Percutaneous drainage (n=22)** | **Laparotomy without pancreatectomy**  **(n=23)** | **Upfront pancreatectomy (n=29)** | **P value** |
| --- | --- | --- | --- | --- |
| Donor age (years) (IQR) | 26 (20-36) | 22 (19-36) | 21.5 (17-30) | 0.39 |
| Donor BMI (kg/m^2^) (IQR) | 22.7 (19.9-26.2) | 24.9 (20.1-28.9) | 23.9 (19.2-27.8) | 0.52 |
| DBD donor (%) | 21 (95.5) | 22 (95.7) | 29 (100) | 0.51 |
| Donor (DCD) WIT (mins) (IQR) | 10 (10-10) | 10 (10-10) | NA | NA |
| Donor CIT (mins) (IQR) | 485 (398-547) | 511 (412-641) | 537.5 (489.2-615.5) | 0.21 |
| Recipient age (years) (IQR) | 41 (36.3-46.1) | 42.7 (40.4-47.6) | 40 (35.9-47.1) | 0.49 |
| Recipient gender (% males) | 16 (72.7) | 10 (43.5) | 21 (72.4) | 0.06 |
| Recipient BMI (kg/m^2^) (IQR) | 26.1 (24.4-28.5) | 25.9 (22.9-29.9) | 25.5 (22.3-28.3) | 0.75 |
| **Recipient CMV status:** | | | | 0.11 |
| CMV mismatch (D+/R-) (%) | 2 (9.1) | 2 (8.7) | 8 (27.6) |  |
| CMV infection (R+) (%) | 3 (13.6) | 1 (4.3) | 5 (17.2) |  |
| **Recipient EBV status:** | | | |  |
| EBV mismatch (D+/R-) (%) | 0 (0) | 0 (0) | 2 (6.9) | 0.20 |
| **Transplant category:** | | | |  |
| SPK (%) | 19 (86.4) | 16 (69.6) | 18 (62.1) | 0.19 |
| PAK (%) | 3 (13.6) | 6 (26.1) | 11 (37.9) |  |
| PTA (%) | 0 (0) | 1 (4.3) | 0 (0) |  |
| Pre-transplant IS (%) | 0 (0) | 0 (0) | 4 (13.8) | 0.04 |
| Pre-transplant dialysis (%) | 9 (40.9) | 5 (21.7) | 11 (37.9) | 0.33 |
| Pre-transplant cardiac intervention (%) | 5 (22.7) | 10 (43.5) | 12 (41.4) | 0.27 |
| Pre-transplant infections (%) | 2 (9.1) | 3 (13) | 3 (10.3) | 0.91 |
| Post-transplant dialysis (%) | 0 (0) | 2 (8.7) | 2 (6.9) | 0.39 |
| Post-transplant stay (days) (IQR) | 9.2 (7.9-13) | 12.5 (8.7-29.2) | 11.6 (8.7-17.6) | 0.21 |
| Post-transplant ICU stay (days) (IQR) | 0 (0-1) | 0 (0-1) | 0 (0-1) | 0.96 |
| **Graft related Complications:** | | | | |
| Very early leak (90 days) (%) | 10 (45.5) | 8 (34.8) | 13 (44.8) | 0.71 |
| Early leak (90 days to 1 year) (%) | 6 (27.3) | 6 (26.1) | 7 (24.1) | 0.67 |
| Delayed leak (beyond 1 year) (%) | 6 (27.3) | 9 (39.1) | 9 (31) | 0.78 |
| Portal vein thrombosis (%) | 1 (4.5) | 0 (0) | 1 (3.4) | 0.61 |
| Hemorrhage (%) | 1 (4.5) | 2 (8.6) | 2 (6.8) | 0.56 |
| Arterial thrombosis (%) | 0 (0) | 1 (4.3) | 0 (0) | 0.32 |
| Graft rejection (pancreas) (%) | 4 (18.1) | 3 (13) | 5 (17.2) | 0.77 |
| Graft rejection (Kidney) (%) | 5 (22.7) | 4 (17.4) | 3 (10.3) | 0.07 |
| Graft loss (Kidney)(%) | 2 (9.1) | 4 (17.4) | 3 (10.3) | 0.12 |
| Re-laparotomy (%) | 2 (9.1) | 3 (13) | 2 (6.9) | 0.78 |
| Re-transplantation (%) | 1 (4.5) | 2 (8.7) | 12 (41.4) | 0.001 |
| Overall mortality (%) | 3 (13.6) | 3 (13) | 8 (27.6) | 0.31 |

** All continuous variables expressed as medians, unless specified otherwise

*Legends: IQR: Interquartile range, DBD: Donation after brain death, BMI: Body mass index, DCD: Donation after cardiac death, WIT: Warm ischemia time, CIT: Cold ischemia time, CMV: Cytomegalovirus, EBV: Epstein Barr Virus, D/R: Donor/Recipient, SPK: Simultaneous pancreas kidney, PAK: Pancreas after kidney, PTA: Pancreas transplant alone, IS: Immunosuppression, ICU: Intensive care unit.*
