# Supplementary figures and images for: Evolving Trends in the Management of Duodenal Leaks After Pancreas Transplantation: A Single-Centre Experience
Source: Transpl Int. 2024 Sep 23;37:13302. doi: 10.3389/ti.2024.13302 (PMC11456492; doi:10.3389/ti.2024.13302)

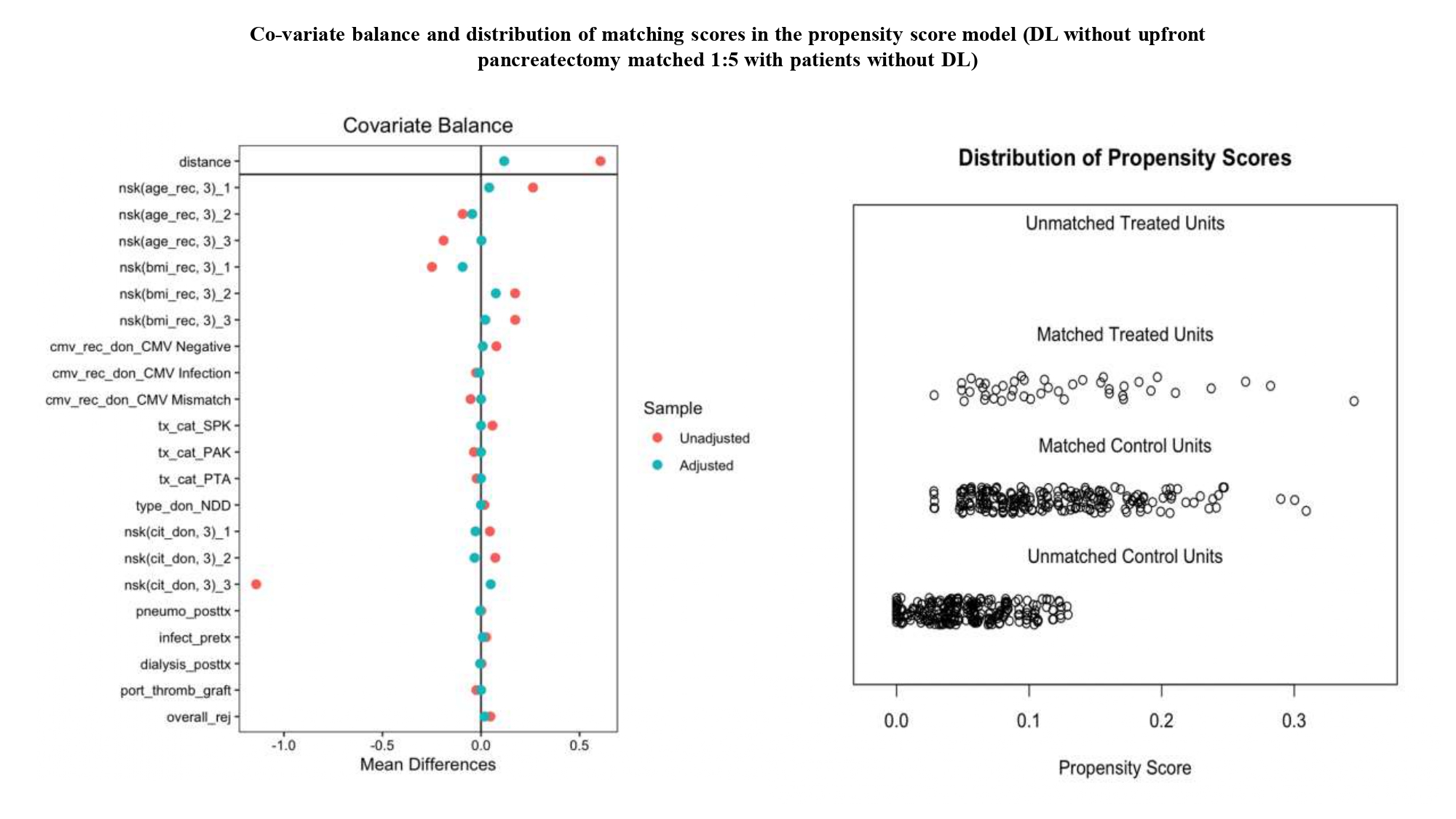

Supplement: Supplementary file 2 [file Image1.JPEG]
