# Supplementary material for: Evolving Trends in the Management of Duodenal Leaks After Pancreas Transplantation: A Single-Centre Experience
Source: Transpl Int. 2024 Sep 23;37:13302. doi: 10.3389/ti.2024.13302 (PMC11456492; doi:10.3389/ti.2024.13302)
Supplement: Supplementary file 3 [file Table2.docx]

**Supplementary table 2:** Cox proportional hazards model in the whole population (n=595) to identify the predictors of overall survival (OS), graft survival (GS) and disease (duodenal leak) free survival (DLFS) (Full model).

| **Variable** | **OS** | | | **GS** | | | **DLFS** | | |
| --- | --- | --- | --- | --- | --- | --- | --- | --- | --- |
|  | **HR** | **95% CI** | **P val** | **HR** | **95% CI** | **P val** | **HR** | **95% CI** | **P val** |
| **Donor age (yrs)** | | | | | | | | | |
| ≤19 | 0.83 | 0.35-1.94 | 0.66 | 1.13 | 0.63-2.04 | 0.68 | 1.05 | 0.54-2.03 | 0.88 |
| 19-34 | 1.08 | 0.49-2.39 | 0.85 | 0.99 | 0.57-1.71 | 0.96 | 0.85 | 0.42-1.71 | 0.65 |
| >34 | 1.00 | 0.42-2.40 | >0.99 | 0.85 | 0.45-1.62 | 0.63 | 1.03 | 0.47-2.28 | 0.93 |
| **Donor BMI (kg/m^2^)** | | | | | | | | | |
| ≤20.8 | 1.02 | 0.53-1.98 | 0.94 | 0.84 | 0.52-1.35 | 0.46 | 0.51 | 0.31-0.82 | 0.005 |
| 20.8-26.1 | 0.64 | 0.31-1.31 | 0.22 | 1.06 | 0.66-1.72 | 0.79 | 0.80 | 0.45-1.40 | 0.43 |
| >26.1 | 0.90 | 0.44-1.83 | 0.76 | 1.23 | 0.72-2.10 | 0.44 | 0.94 | 0.48-1.85 | 0.86 |
| DBD vs DCD donor | 0.51 | 0.17-1.56 | 0.24 | 1.33 | 0.53-3.35 | 0.54 | 2.15 | 0.55-8.47 | 0.27 |
| **Donor CIT (mins)** | | | | | | | | | |
| ≤442.2 | 0.65 | 0.33-1.29 | 0.22 | 0.99 | 0.62-1.59 | 0.98 | 0.94 | 0.40-2.25 | 0.89 |
| 442.2-644.7 | 1.11 | 0.61-2.03 | 0.74 | 1.02 | 0.66-1.58 | 0.91 | 1.02 | 0.49-2.10 | 0.96 |
| >644.7 | 1.12 | 0.56-2.25 | 0.74 | 1.05 | 0.66-1.68 | 0.83 | 0.36 | 0.12-1.02 | 0.06 |
| **Recipient age (yrs)** | | | | | | | | | |
| ≤37.3 | 1.06 | 0.26-4.28 | 0.93 | 0.75 | 0.49-1.16 | 0.19 | 1.36 | 0.60-3.08 | 0.46 |
| 37.3-50.5 | 2.21 | 0.65-7.57 | 0.21 | 0.75 | 0.48-1.17 | 0.20 | 0.92 | 0.42-2.02 | 0.83 |
| >50.5 | 4.14 | 1.13-15.2 | **0.03** | 0.89 | 0.51-1.54 | 0.68 | 1.09 | 0.42-2.83 | 0.85 |
| **Recipient BMI (kg/m^2^)** | | | | | | | | | |
| ≤21.8 | 0.46 | 0.23-0.95 | **0.03** | 0.48 | 0.24-0.84 | 0.01 | 1.38 | 0.59-3.22 | 0.46 |
| 21.8-27.9 | 0.45 | 0.22-0.93 | **0.03** | 0.48 | 0.27-0.87 | 0.01 | 1.99 | 0.89-4.22 | 0.09 |
| >27.9 | 0.78 | 0.37-1.65 | 0.51 | 0.77 | 0.41-1.47 | 0.43 | 1.98 | 0.84-4.63 | 0.12 |
| Male recipient | 0.78 | 0.49-1.24 | 0.28 | 1.30 | 0.91-1.84 | 0.15 | 0.95 | 0.60-1.49 | 0.81 |
| CMV mismatch (D+/R-) | 1.08 | 0.55-2.13 | 0.82 | 1.13 | 0.76-1.66 | 0.55 | 1.06 | 0.58-1.91 | 0.86 |
| CMV infection (R+) | 0.76 | 0.38-1.50 | 0.43 | 1.49 | 1.01-2.21 | **0.04** | 0.99 | 0.50-1.98 | 0.99 |
| EBV mismatch (D+/R-) | 0.60 | 0.12-2.95 | 0.53 | 1.44 | 0.79-2.61 | 0.23 | 0.28 | 0.03-2.96 | 0.29 |
| EBV infection (R+) | 2.58 | 0.37-17.9 | 0.34 | 0.00 | 0.00-0.00 | <0.001 | 0.00 | 0.00-0.00 | <0.001 |
| PAK vs SPK | 1.07 | 0.57-1.94 | 0.82 | 1.61 | 1.08-2.38 | **0.02** | 1.00 | 0.58-1.74 | >0.99 |
| PTA vs SPK | 0.93 | 0.17-5.26 | 0.94 | 1.32 | 0.50-3.46 | 0.58 | 0.40 | 0.06-2.83 | 0.36 |
| Pre-transplant IS | 1.07 | 0.37-3.08 | 0.89 | 0.86 | 0.52-1.40 | 0.54 | 0.71 | 0.24-2.07 | 0.53 |
| Pre-transplant dialysis | 0.88 | 0.54-1.46 | 0.63 | 1.10 | 0.78-1.55 | 0.59 | 0.89 | 0.55-1.42 | 0.62 |
| Pre-transplant infection | 1.58 | 0.79-3.17 | 0.19 | 1.66 | 1.10-250 | **0.02** | 1.45 | 0.73-2.90 | 0.29 |
| Pre-transplant cardiac intervention | 1.40 | 0.86-2.26 | 0.17 | 0.84 | 0.61-1.17 | 0.31 | 0.84 | 0.53-1.35 | 0.48 |
| Post-transplant dialysis | 2.29 | 0.94-5.59 | 0.07 | 1.80 | 1.11-2.94 | **0.02** | 1.01 | 0.40-2.54 | 0.98 |
| Post-transplant pneumonia | 3.08 | 1.32-7.18 | **0.009** | 1.38 | 0.69-2.75 | 0.36 | 2.37 | 0.78-7.20 | 0.13 |
| Post-transplant DVT | 2.83 | 1.32-6.09 | **0.008** | 0.00 | 0.00-0.00 | <0.001 | 3.51 | 0.71-17.4 | 0.12 |
| Post-transplant CLABSI | 0.00 | 0.00-0.00 | <0.001 | 2.84 | 1.55-5.23 | **<0.001** | 0.00 | 0.00-0.00 | <0.001 |
| Graft arterial thrombosis | 0.00 | 0.00-0.00 | <0.001 | 2.01 | 0.92-4.39 | 0.08 | 1.91 | 0.51-7.14 | 0.34 |
| Graft portal vein thrombosis | 0.92 | 0.18-4.76 | 0.92 | 1.63 | 0.73-3.64 | 0.23 | 0.00 | 0.00-0.00 | <0.001 |
| Duodenal leak | 0.51 | 0.07-3.56 | 0.49 | 3.27 | 1.98-5.39 | **<0.001** | - | - | - |
| Graft rejection (Pancreas) | 1.22 | 0.58-2.60 | 0.60 | 1.64 | 1.16-2.33 | **0.005** | 1.08 | 0.57-2.06 | 0.81 |

*Legends: OS: Overall survival, GS: Graft survival, DLFS: Duodenal leak free survival, HR: Hazard ratio, CI: Confidence interval, DBD: Donation after brain death, DCD: Donation after cardiac death, BMI: Body mass index, CIT: Cold ischemia time, CMV: Cytomegalovirus, EBV: Epstein Barr Virus, D/R: Donor/Recipient, SPK: Simultaneous pancreas kidney, PAK: Pancreas after kidney, PTA: Pancreas transplant alone, IS: Immunosuppression, DVT: Deep venous thrombosis, CLABSI: Central line associated bloodstream infections*

*Note: Donor age, BMI, CIT and recipient age and BMI (Non-linear variables) were transformed using cubic spline functions with 3 degrees of freedom; knots are placed at the 25th and 75th percentiles of the variable in the overall population*
